# Detoxifying *Escherichia coli* for endotoxin-free production of recombinant proteins

**DOI:** 10.1186/s12934-015-0241-5

**Published:** 2015-04-16

**Authors:** Uwe Mamat, Kathleen Wilke, David Bramhill, Andra Beate Schromm, Buko Lindner, Thomas Andreas Kohl, José Luis Corchero, Antonio Villaverde, Lana Schaffer, Steven Robert Head, Chad Souvignier, Timothy Charles Meredith, Ronald Wesley Woodard

**Affiliations:** Division of Structural Biochemistry, Research Center Borstel, Leibniz-Center for Medicine and Biosciences, Parkallee 1-40, D-23845 Borstel, Germany; Research Corporation Technologies, Inc, 5210 East Williams Circle, Suite 240, Tucson, AZ 85711-4410 USA; Division of Immunobiophysics, Research Center Borstel, Leibniz-Center for Medicine and Biosciences, Parkallee 1-40, D-23845 Borstel, Germany; Division of Bioanalytical Chemistry, Research Center Borstel, Leibniz-Center for Medicine and Biosciences, Parkallee 1-40, D-23845 Borstel, Germany; Division of Molecular Mycobacteriology, Research Center Borstel, Leibniz-Center for Medicine and Biosciences, Parkallee 1-40, D-23845 Borstel, Germany; CIBER de Bioingeniería, Biomateriales y Nanomedicina (CIBER-BBN), Bellaterra, 08193 Cerdanyola del Vallès, Spain; Institut de Biotecnologia i de Biomedicina, Universitat Autònoma de Barcelona, Bellaterra, 08193 Cerdanyola del Vallès, Spain; Departament de Genètica i de Microbiologia, Universitat Autònoma de Barcelona, Bellaterra, 08193 Cerdanyola del Vallès, Spain; NGS and Microarray Core Facility, The Scripps Research Institute, 10550 North, Pines Road, La Jolla, Torrey, CA 92037 USA; Department of Biochemistry and Molecular Biology, 206 South Frear, Pennsylvania State University, University Park, PA 16802 USA; Department of Medicinal Chemistry, University of Michigan, 428 Church Street, Ann Arbor, MI 48109-1065 USA; Present address: Bramhill Biological Consulting, LLC, 8240 East Moonstone Drive, Tucson, AZ 85750 USA

**Keywords:** *Escherichia coli*, Lipopolysaccharide, Lipid A, Endotoxic activity, Recombinant protein, TLR4/MD-2 activation

## Abstract

**Background:**

Lipopolysaccharide (LPS), also referred to as endotoxin, is the major constituent of the outer leaflet of the outer membrane of virtually all Gram-negative bacteria. The lipid A moiety, which anchors the LPS molecule to the outer membrane, acts as a potent agonist for Toll-like receptor 4/myeloid differentiation factor 2-mediated pro-inflammatory activity in mammals and, thus, represents the endotoxic principle of LPS. Recombinant proteins, commonly manufactured in *Escherichia coli*, are generally contaminated with endotoxin. Removal of bacterial endotoxin from recombinant therapeutic proteins is a challenging and expensive process that has been necessary to ensure the safety of the final product.

**Results:**

As an alternative strategy for common endotoxin removal methods, we have developed a series of *E. coli* strains that are able to grow and express recombinant proteins with the endotoxin precursor lipid IV_A_ as the only LPS-related molecule in their outer membranes. Lipid IV_A_ does not trigger an endotoxic response in humans typical of bacterial LPS chemotypes. Hence the engineered cells themselves, and the purified proteins expressed within these cells display extremely low endotoxin levels.

**Conclusions:**

This paper describes the preparation and characterization of endotoxin-free *E. coli* strains, and demonstrates the direct production of recombinant proteins with negligible endotoxin contamination.

**Electronic supplementary material:**

The online version of this article (doi:10.1186/s12934-015-0241-5) contains supplementary material, which is available to authorized users.

## Background

In the last thirty years, the biopharmaceutical industry has brought more than 220 biologics to market, giving rise to sales of approximately $70 - $80 billion per year [1]. One third of the unique recombinant protein therapeutics [2] and approximately one half of all products approved [3] are produced using an *Escherichia coli* based expression platform. However, the outer membrane of *E. coli*, like that of most Gram-negative bacteria, contains the potent immunostimulatory molecule lipopolysaccharide (LPS). In mammalian hosts, LPS (also known as endotoxin) can induce a pyrogenic response and ultimately trigger septic shock. Contaminating LPS must therefore be removed from recombinant therapeutic proteins expressed in *E. coli* before they can be safely administered to human patients. The removal of endotoxin from recombinant therapeutics and the testing to demonstrate endotoxin levels below a minimal threshold requires considerable effort, adding significant developmental and manufacturing costs. To date, no post-expression methodologies have been described that can remove endotoxin entirely [4]. Common endotoxin removal methods, such as ultrafiltration, Triton X phase separation, anion-exchange chromatography, adsorption on activated carbon, or treatment with polymyxin B- or histamine-immobilized affinity resins, are plagued by low efficiency and unsatisfactory selectivity [5]. In this context, it is important to note that commercially available recombinant proteins manufactured in *E. coli* may contain residual endotoxin in low but still sufficient quantities to activate human immune cells [6].

Cells of the innate immune system mediate the endotoxic response in mammals. LPS-mediated activation of a cell-surface receptor, consisting of Toll-like receptor 4 (TLR4) complexed with myeloid differentiation factor 2 (MD-2), results in the production of pro-inflammatory cytokines and type-1 interferons that are the chief effectors of the endotoxic response [7]. Research relating the structure of LPS to the activation of TLR4/MD-2 has demonstrated that lipid A is the component of LPS that is responsible for its TLR4/MD-2-dependent endotoxic activity [8]. Alterations in the structure of lipid A, chiefly modifications to the structure, number, and attachment sites of the hydrophobic acyl chains to the di-glucosamine backbone, significantly impacts endotoxic activity through altering TLR4/MD-2 mediated signaling [9,10]. When all the secondary acyl chains are removed, the under-acylated lipid A precursor lipid IV_A_ not only lacks endotoxic activity in human immune cells, but also becomes a hTLR4/MD-2 receptor antagonist [8].

Until recently, it was believed that the structural features of LPS required to maintain the integrity of the Gram-negative outer membrane were essentially the same as the structural features of LPS required to elicit an endotoxic immune response in mammalian cells. The minimal structure essential for survival of typical laboratory strains of *E. coli* K-12 was thought to consist of a molecule of lipid A glycosylated at the 6′ position with two 3-deoxy-d-*manno*-oct-2-ulosonic acid (Kdo) sugar residues. This view changed when we reported the construction and characterization of KPM22, a derivative of the *E. coli* K-12 wild-type strain BW30270 that is unable to synthesize Kdo and yet retains viability with lipid IV_A_ as the predominant outer membrane LPS component [11]. Subsequent research identified gain of function suppressor mutations in the LPS transport apparatus that apparently promote flipping of lipid IV_A_ across the inner membrane [12]. These mutations both remove toxic side effects normally associated with lipid IV_A_ accumulation in the inner membrane as well as provide a sufficient concentration of lipid IV_A_ to support outer membrane biogenesis. The discovery of KPM22 presented us with the opportunity to construct recombinant protein expression strains of *E. coli* with low intrinsic endotoxic potential by rationally reprogramming the outer membrane biosynthesis pathway to elaborate solely lipid IV_A_.

This report describes the construction of stable *E. coli* strains, including derivatives of the popular expression strain BL21 (DE3), capable of efficiently expressing recombinant proteins which are essentially free of endogenous endotoxin contamination under standard laboratory conditions. To evaluate the utility of this expression platform, we applied it to the production of two different human proteins: apolipoprotein A-1 (ApoA-1) and heat shock protein 70 (Hsp70), both known to avidly bind endotoxin, and demonstrate marked reduction in endotoxin activity from minimally purified recombinant proteins.

## Results and discussion

### Engineering of LPS biosynthesis in *E. coli*

**Figure 1:**
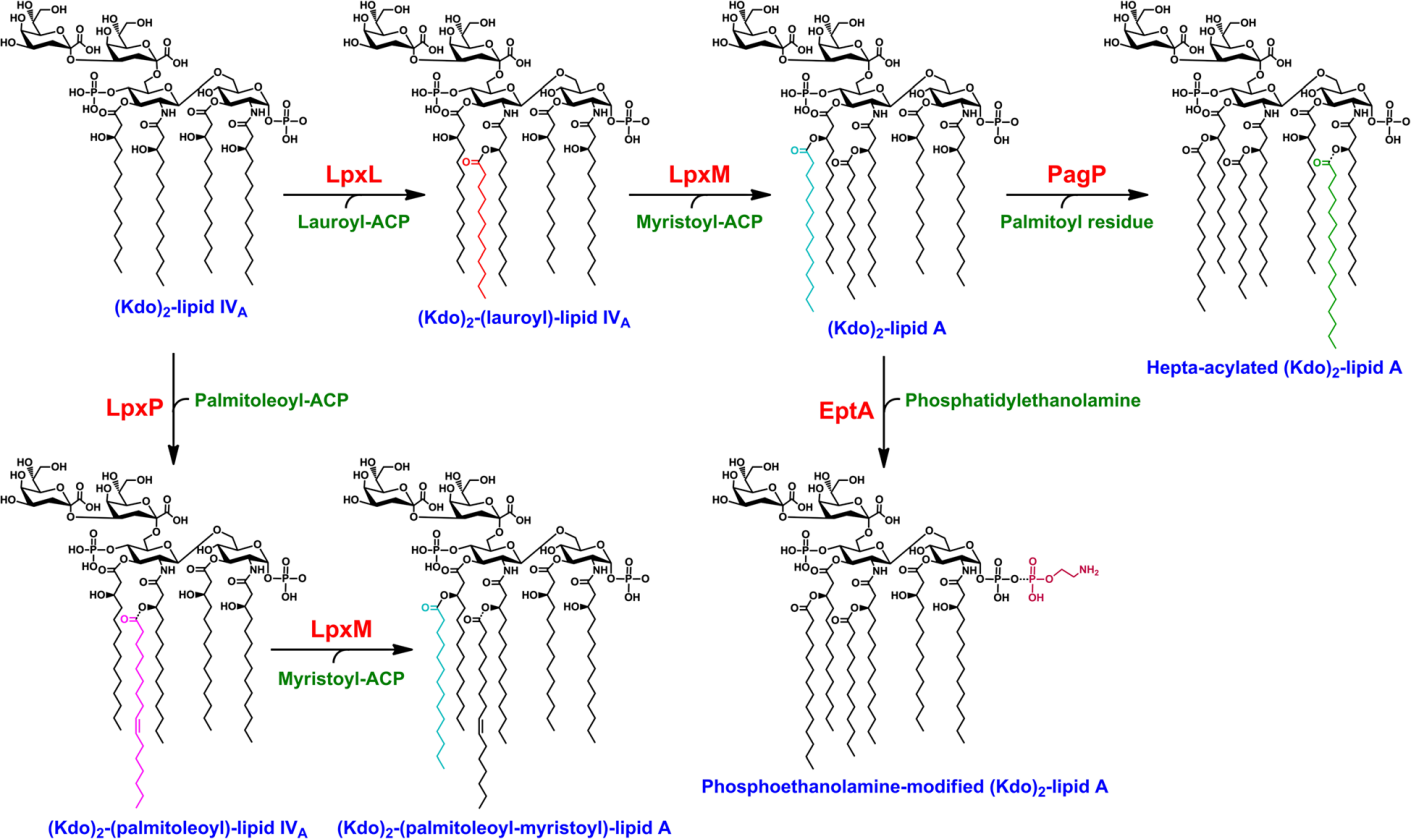
Biosynthetic reactions targeted during the construction of *E. coli* strains KPM318 and KPM404. The late acyltransferases LpxL and LpxM of the constitutive pathway transfer laurate and myristate, respectively, to Kdo_2_-lipid IV_A_ to form the characteristic acyloxyacyl units of hexaacylated Kdo_2_-lipid A. In contrast, LpxP, PagP and EptA are regulated in response to certain stimuli such as incorporation of palmitoleate in place of laurate by LpxP at low growth temperatures or PagP-catalyzed palmitoylation of lipid A upon phospholipid translocation to the outer leaflet of the outer membrane, for example, in strains defective in LPS biosynthesis. The acyl-acyl carrier protein (ACP) serves as the preferred acyl donor for various lipid A-acyltransferases.

**Figure 2:**
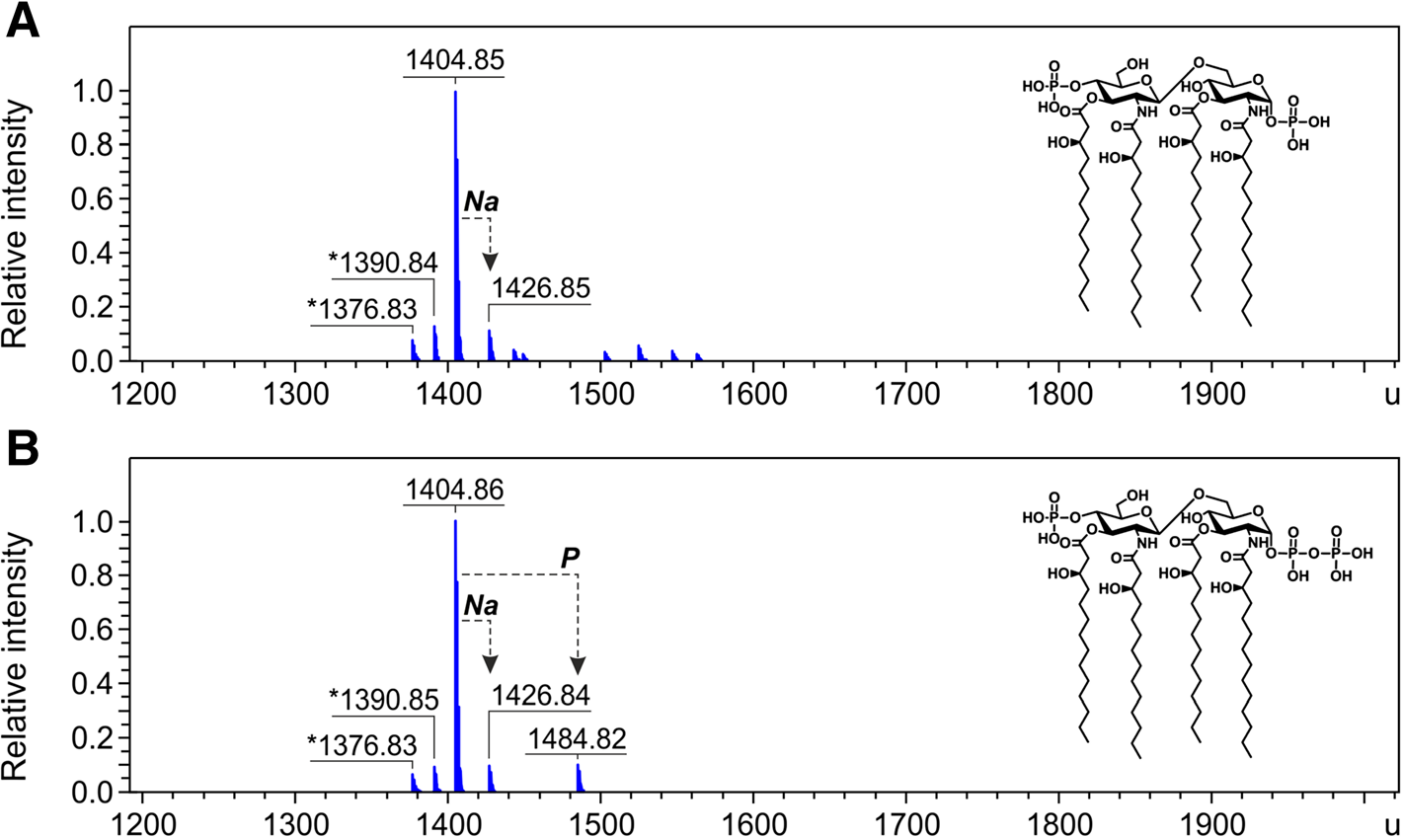
Charge deconvoluted ESI FT-ICR mass spectra in negative ion mode of lipid IV_A_ isolated from BW30270-derived mutants. The lipid IV_A_ (calculated mass 1404.854 u) was extracted from *E. coli* (K-12) strains KPM318 **(A)** and KPM335 **(B)**. Mass numbers given refer to the monoisotopic masses of neutral molecules. Peaks representing molecules with variations in acyl chain length are labeled with asterisks (∆*m* = 14.02 u). The molecular ion at 1484.82 u in panel **B** indicates the presence of a minor fraction of 1-diphosphate lipid IV_A_. The structures of lipid IV_A_ and 1-diphosphate lipid IV_A_ are shown as *insets* in panels **A** and **B**, respectively.

We began our construction of LPS-free *E. coli* K-12 using the Kdo-depleted strain KPM22 L11 [12]. This strain contains deletions of *kdsD* and *gutQ*, which encode d-arabinose 5-phosphate isomerases essential for the biosynthesis of Kdo [13,14], and a C:G to T:A transition at position 52 of *msbA*, which acts as a suppressor of the normally lethal ∆Kdo phenotype [12]. To produce a strain that contains lipid IV_A_ as the only LPS outer membrane component and that cannot revert to synthesize endotoxic derivatives, we sequentially generated mutant strains with unmarked deletions of the genes *lpxL*, *lpxM*, *pagP*, *lpxP* and *eptA*. These genes encode enzymes acting downstream of Kdo incorporation into lipid IV_A_, and are either part of the constitutive pathway of Kdo_2_-hexaacyl lipid A biosynthesis (the Kdo_2_-lipid IV_A_ lauroyl-ACP acyltransferase LpxL and the Kdo_2_-lauroyl-lipid IV_A_ myristoyl-ACP acyltransferase LpxM), modify lipid A with additional acyl chains (the phospholipid:lipid A palmitoyl transferase PagP and the Kdo_2_-lipid IV_A_ palmitoleoyl-ACP acyltransferase LpxP), or append phosphoethanolamine (P-EtN) under certain conditions (the lipid A P-EtN transferase EptA) [15] (Figure 1). Analysis of the LPS isolated from this strain, designated KPM318, using electrospray-ionization Fourier-transformed ion cyclotron mass spectrometry (ESI FT-ICR) revealed a single primary peak at 1404.85 u, consistent with the structure of the unmodified tetraacyl-1,4′-bisphosphate LPS precursor lipid IV_A_ (Figure 2A).

The successful construction of KPM318 demonstrated the viability of *E. coli* containing only lipid IV_A_. However, like other *E. coli* K-12 strains with partially defective outer membranes such as the ∆Kdo prototype strain KPM22 [11], KPM318 displayed growth defects at temperatures above 40°C. To overcome this, we isolated a series of stable temperature-resistant derivatives of KPM318 capable of growing exponentially at 42°C. KPM335 was the most robust of the spontaneous mutants. Whole-genome sequencing of KPM335 identified a single *de novo* mutation in comparison to the temperature sensitive KPM318 parent strain, a G:C to T:A transversion at base number 181 of the *frr* gene (Additional file 1: Table S1). The *frr* gene encodes an essential ribosome recycling factor which has been described to play multiple cellular roles, such as in disassembly of the post-termination complex [16], prevention of translation errors [17], promoting translational coupling [18], and increasing cell viability through polyamine stimulation of stationary-phase *E. coli* cultures [19]. The derivation of KPM335 argues strongly for a direct correlation between the development of the *frr181* allele and the ability of the strain to tolerate the ∆Kdo phenotype at 42°C. However, elucidation of the underlying mechanism must await further investigations.

ESI FT-ICR analysis of the LPS isolated from KPM335 revealed no significant changes in the LPS composition, with lipid IV_A_ remaining the predominant LPS-related molecule as in the parent KPM318 strain (Figure 2B). However, in contrast to KPM318, spectra of KPM335 displayed a minor peak with a molecular mass of 1484.82 u, consistent with the structure of 1-diphosphate lipid IV_A_. Touzé and coworkers have shown previously that transfer of a second phosphate group to the 1 position of lipid A is catalyzed by LpxT, an inner membrane protein of the undecaprenyl-pyrophosphate phosphatase family, which is able to phosphorylate Kdo_2_-lipid IV_A_*in vitro*, but not lipid acceptors lacking Kdo [20]. Thus, the presence of a minor *tris*-phosphorylated lipid IV_A_ fraction in KPM335 argues against an absolute requirement of LpxT for Kdo-glycosylated lipid A acceptors under *in vivo* conditions. It remains unclear, however, why phosphorylation of lipid IV_A_, albeit with a very low efficiency, can occur in KPM335, but apparently not in its KPM318 parent strain.

Apart from the *frr181* mutation in KPM335, a total of 12 mutations were specific to both KPM318 and KPM335, including ∆*kdsD*, ∆*gutQ*, ∆*lpxL*, ∆*lpxM*, ∆*pagP*, ∆*lpxP*, ∆*eptA* and *msbA52* as a prerequisite for synthesis of lipid IV_A_ as the predominant LPS-related outer membrane component. For another four mutations, we believe that they arose spontaneously during the generation of mutant strains, namely a silent mutation located in *yqiI*, two missense mutations in *gor* and *waaY*, and a point mutation in the non-coding region upstream of the deleted *eptA* gene. The latter mutation is most likely a result of KPM274 construction as a donor strain of the Δ*eptA::kan* cassette. The mutation is located within the sequence of the homology region of primer ECOeptAH1 (Additional file 2: Table S2) and suggests an error in PCR amplification of the kanamycin resistance cassette targeting the *eptA* gene and ultimately integration into the genome of KPM318. Compared to the reference genome sequence of the common *E. coli* MG1655 progenitor, the strains BW30270, KPM318 and KPM335 share sequence variations at six locations. Of these, both a silent nucleotide substitution at position 114 and a single nucleotide insertion at base number 253 of *ylbE*, and deletion of nucleotide 151 within the *glpR* gene have been described recently as genetic variations in common *E. coli* MG1655 stock cultures [21]. Furthermore, while *E. coli* MG1655 has been shown to express a defective ribonuclease PH due to a frameshift by deletion of the first nucleotide of codon 223 of the pseudogene *rph-1* [22], insertion of a single nucleotide as the first base of codon 224 of *rph-1* predicts reconstitution of RNase PH function and elimination of the polar effect of the *rph-1* mutation on the downstream *pyrE* gene in BW30270, KPM318 and KPM335.

Next, we decided to replicate the unique set of unmarked genomic deletions in the genetic background of the popular *E. coli* expression strain BL21 (DE3). As a first step towards the construction of an LPS-free *E. coli* BL21 (DE3) derivative, we replaced the wild-type *msbA* gene with the *msbA148* suppressor allele of strain KPM22 L1 [12]. This allowed the resulting strain, MWB03, to tolerate null mutations in otherwise essential genes of the LPS biosynthesis pathway. We then sequentially deleted the same genes that had been deleted during the creation of KPM318. Whole-genome sequencing of the final strain, KPM404, confirmed the presence of the *msbA148* suppressor mutation (Additional file 3: Table S3), and verified the absence of the *kdsD*, *gutQ*, *lpxL*, *lpxM*, *pagP*, *lpxP* and *eptA* genes. In comparison to the genome sequence of *E. coli* BL21 (DE3), we further identified a silent mutation in *yceJ*, three missense changes within the coding sequences of YadG, ECD_00513 and RstA, and a point mutation in the intergenic region between *nudC* and *hemE*. Finally, a total of five single nucleotide substitutions were counted in the region between nucleotides 46 and 222 of the *basR* gene downstream of ∆*eptA*. These substitutions perfectly matched the sites of *basR* sequence variations in *E. coli* B and K-12, indicating that about one third of the BasR-encoding gene of KPM404 was replaced by the corresponding *basR* sequence of *E. coli* K-12. Just as for the construction of KPM318, the *E. coli* K-12 strain KPM274 served as the donor for transfer of the Δ*eptA::kan* cassette via P1*vir* transduction to yield strain KPM403, which well explains the generation of a *basR* hybrid sequence by co-transduction of the Δ*eptA::kan* cassette and adjacent *basR* sequences. In fact, as described above, the use of KPM274 as a Δ*eptA::kan* donor strain also explains why KPM404 carries a point mutation at the same position upstream of the deleted *eptA* gene as in KPM318.

**Figure 3:**
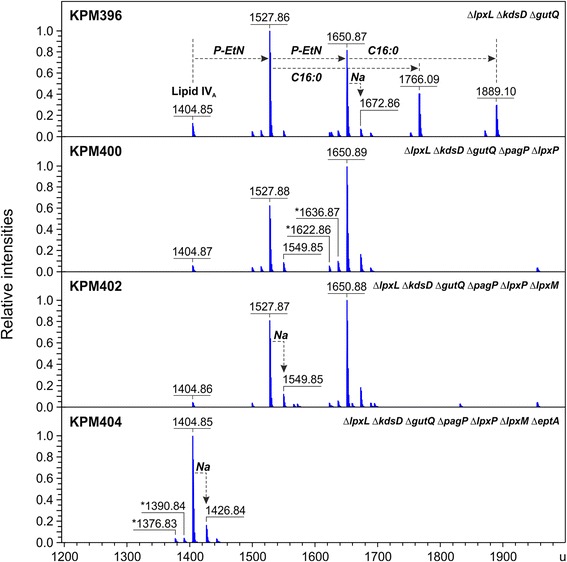
Charge deconvoluted ESI FT-ICR mass spectra in negative ion mode of LPS isolated from BL21 (DE3)-derived mutants. Mass numbers given refer to the monoisotopic masses of neutral molecules. Peaks representing molecules with variations in acyl chain length are labeled with asterisks (∆*m* = 14.02 u). The mass spectra depict the progress in elimination of lipid IV_A_ heterogeneity by sequential deletion of genes encoding the addition of acyl chains and P-EtN to the lipid A precursor.

**Figure 4:**
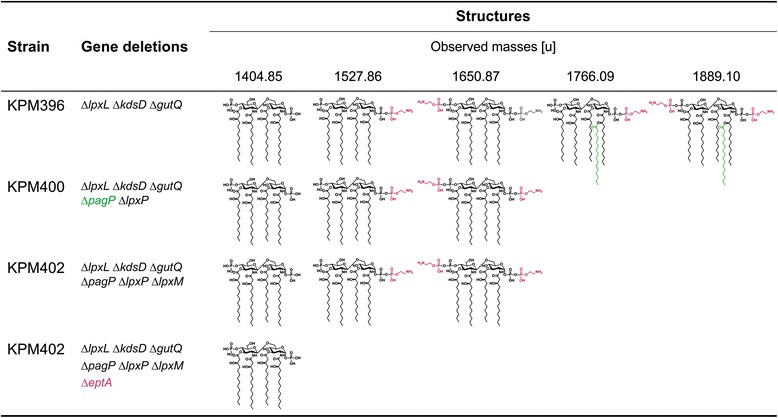
Structures and molecular masses of lipid IV_A_ molecules identified by ESI FT-ICR mass spectrometry in BL21 (DE3)-derived KPM mutants. The ESI FT-ICR mass spectra are shown in Figure 3. Modifications of lipid IV_A_ with palmitate (green) and P-EtN (magenta) are catalyzed by PagP and EptA, respectively.

Mass spectrometric analyses of the LPS profiles from mutants established in the BL21 (DE3) genetic background underscored the need for radical modification of LPS biosynthesis to accomplish synthesis of only lipid IV_A_ in KPM404 (Figures 3 and 4). While disruption of the *gutQ*, *kdsD* and *lpxL* genes in the intermediate mutant strain KPM396 resulted in synthesis of non-glycosylated lipid IV_A_ precursors lacking the secondary lauroyl and myristoyl chains, mass spectrometry revealed a rather heterogeneous mixture of differently modified lipid IV_A_ species. The spectra displayed four prominent peaks with molecular masses of lipid IV_A_ substituted with one P-EtN group (1527.86 u), lipid IV_A_ modified with two P-EtN moieties (1650.87 u), and palmitoylated lipid IV_A_ molecules carrying either one (1766.09 u) or two (1889.10 u) P-EtN residues. Since lipid A palmitoylation seems to be an indication for an adaptive response to aberrant translocation of phospholipids to the outer leaflet of the outer membrane [23], we suspect that PagP-mediated transfer of palmitate to lipid IV_A_ is triggered by perturbations of outer membrane lipid asymmetry in Kdo-depleted strains of the KPM series. As shown for the LPS sample of KPM400, complete loss of the palmitoylated lipid IV_A_ fraction was achieved by deletion of the *pagP* gene, leaving lipid IV_A_ molecules modified with either one or two P-EtN groups unaffected.

Due to the block in Kdo biosynthesis and the lack of LpxL, Kdo_2_-lipid IV_A_ and Kdo_2_-(lauroyl)-lipid IV_A_, the preferred substrates for LpxP and LpxM, respectively [24-26], cannot be synthesized in strains derived from KPM396. In addition, previous work has shown that expression of LpxP is induced under conditions of cold shock (12°C) to incorporate an unsaturated C16:1 acyl chain at the expense of a laurate (C12:0), perhaps reflecting the demand to adjust membrane fluidity in the cold [25,27]. It was therefore not surprising that deletion of the *lpxP* and *lpxM* genes did not exhibit an obvious effect on lipid IV_A_ composition of KPM400 and KPM402, respectively. There is no data indicating that LpxP and LpxM are capable of using lipid IV_A_ as an acceptor substrate. It seems to be quite possible, however, that both enzymes show low levels of activity under specific conditions. Contrary to the proposed physiological role of LpxP in adaptation of *E. coli* cells to low growth temperatures, limited induction of LpxP expression has been demonstrated as a potential compensatory mechanism even at 30°C in *lpxL* and *lpxL lpxM* mutants of *E. coli* W3110 [28]. Likewise, LpxM was able to transfer a myristoyl chain directly to Kdo_2_-lipid IV_A_ in *E. coli* W3110 strains lacking *lpxL* and *lpxL lpxP* [28].

Like PagP and LpxP, EptA-dependent modification of lipid A with P-EtN is part of the complex regulatory network associated with the structural redesign of LPS upon exposure to changing environmental conditions or envelope stress factors. As shown for *E. coli* K-12, modification of lipid A with P-EtN occurs under certain conditions such as in response to external stimuli like ammonium metavanadate [29] or mild acid pH [30]. Although P-EtN appears to be transferred predominantly to the 1-phosphate group of lipid A [31], double P-EtN substitutions at the 1- und 4′-phosphate positions were evident in lipid A of *E. coli* K-12 lacking LpxT activity [32] and lipid IV_A_ of a mutant strain defective in MsbA-dependent translocation of LPS across the inner membrane [12]. Based upon ESI FT-ICR analysis presented here, deletion of the *eptA* gene was clearly necessary as well as sufficient to prevent lipid IV_A_ of KPM404 from being substituted with one or two P-EtN residues. Thus, our data not only corroborate previous findings on the ability of EptA to transfer P-EtN to both the 1- and the 4′-phosphate group of lipid A [32], but also provide experimental evidence for its ability to use lipid IV_A_ as a substrate for single and double P-EtN modifications.

In contrast to the *E. coli* K-12 strain KPM318, integration of lipid IV_A_ into the outer membrane of KPM404 did not result in a temperature-sensitive phenotype of the BL21 (DE3)-derived mutant (data not shown). Although closely related at the genomic level [33], combined analysis of the genomes, transcriptomes, proteomes and phenomes of *E. coli* K-12 and BL21 (DE3) revealed significant differences in their cellular physiology and metabolism [34], which may explain the differences in the ability of KPM318 and KPM404 to maintain outer membrane integrity in the presence of lipid IV_A_ at temperatures above 40°C.

### Biological activity of engineered *E. coli* cells and LPS

**Figure 5:**
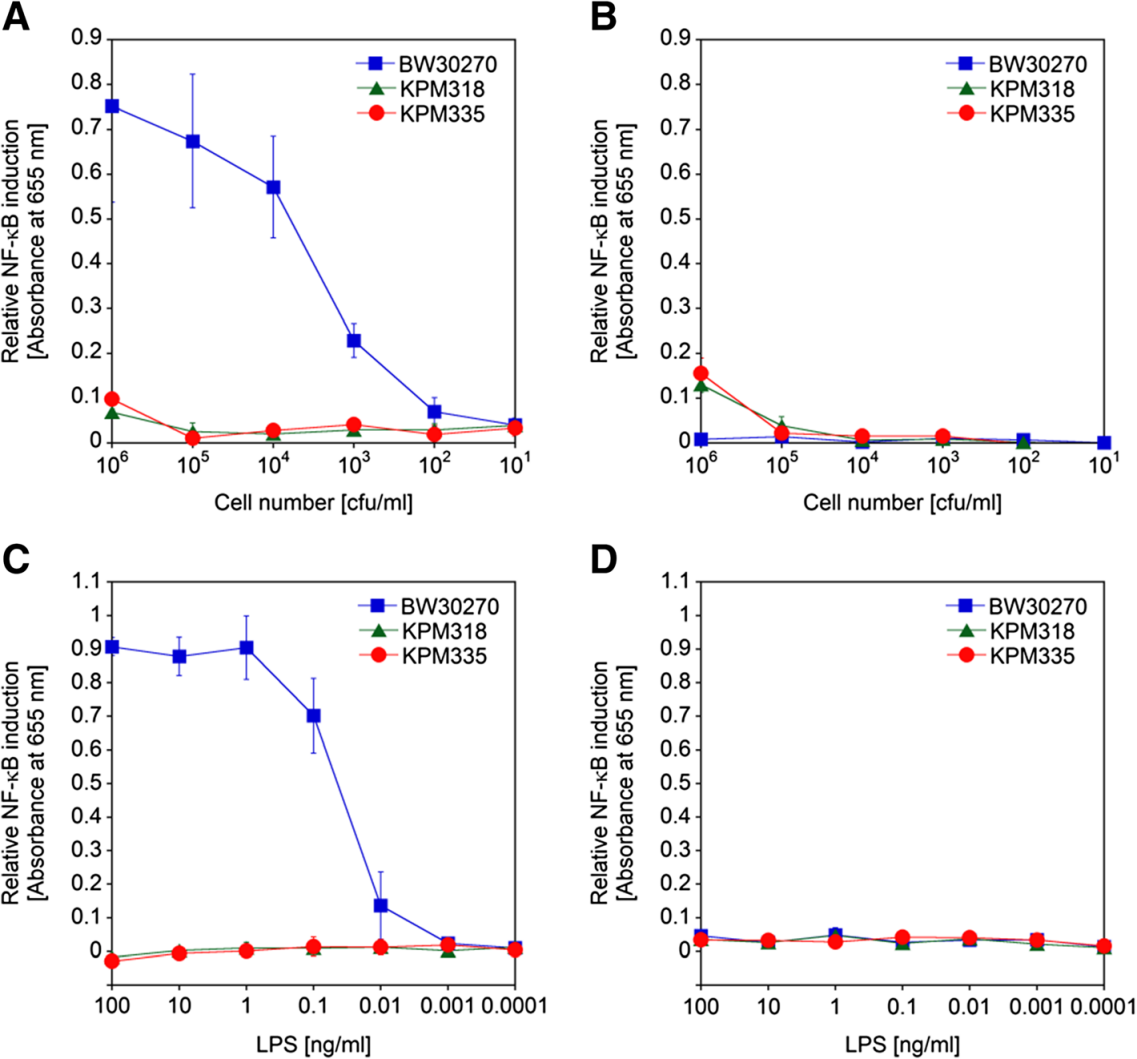
Dose–response curves of NF-κB induction by whole bacterial cells and LPS of *E. coli* K-12 strains. The samples were assayed with HEK-Blue hTLR4 cells for hTLR4/MD-2-mediated NF-κB induction by colorimetric determination of NF-κB-dependent SEAP activity **(A** **and** **C)**. HEK-Blue Null2 cells, the parental cell line of HEK-Blue hTLR4 cells lacking the hTLR4/MD-2 receptor complex, were used as a control **(B** **and** **D)**. HEK-Blue hTLR4 and Null2 cells were stimulated with tenfold serial dilutions of whole bacterial cells **(A** **and** **B)** and LPS extracts **(C** **and** **D)** of KPM318 and KPM335 in comparison to their parental strain BW30270, respectively. The values represent the means and standard deviations from three individual experiments. In all experiments, assayed samples showed low level stimulation of HEK-Blue Null2 cells, indicating that NF-κB-dependent SEAP expression was specifically induced via the hTLR4/MD-2 signaling pathway in HEK-Blue hTLR4 cells.

**Figure 6:**
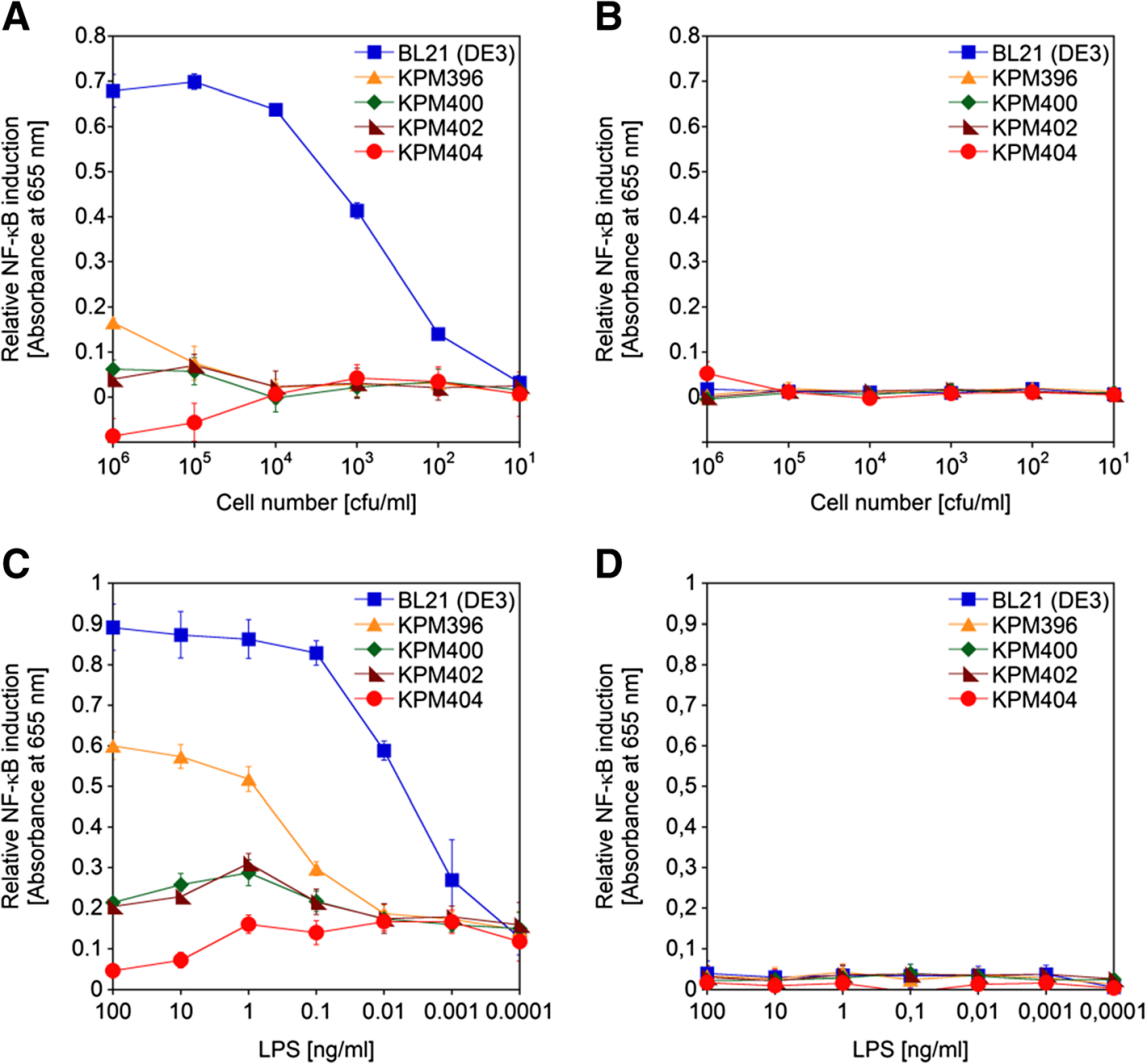
Dose–response curves of NF-κB induction by whole bacterial cells and LPS of *E. coli* BL21 (DE3) strains. The samples were assayed with HEK-Blue hTLR4 **(A** **and** **C)** and Null2 **(B** **and** **D)** cells for relative NF-κB induction by colorimetric determination of NF-κB-dependent SEAP activity. Relative NF-κB induction was measured following stimulation of HEK-Blue hTLR4 and Null2 cells with tenfold serial dilutions of whole bacterial cells **(A** **and** **B)** and LPS extracts **(C** **and** **D)** of *E. coli* BL21 (DE3) and BL21 (DE3)-derived KPM mutants, respectively. The values represent the means and standard deviations from three individual experiments. In all experiments, assayed samples displayed negligible activation of parental HEK-Blue Null2 cells, suggesting specific induction of NF-κB-dependent SEAP expression via the hTLR4/MD-2 signaling pathway in HEK-Blue hTLR4 cells.

To test the endotoxic potential of the engineered *E. coli* K-12 and B strains, we performed TLR4/MD-2 activation assays using HEK-Blue hTLR4 cells. Stimulation of these cells, which express human TLR4, MD-2 and CD14 on their surfaces, induces the production of the NF-κB- and activator protein-1 (AP-1)-dependent reporter secreted embryonic alkaline phosphatase (SEAP). The phosphatase levels can be determined by reading the absorbance at 655 nm using a colorimetric substrate. In order to address the question of whether NF-κB is specifically induced via the hTLR4/MD-2 signaling pathway, HEK-Blue Null2 cells, the parental cell line of HEK-Blue hTLR4 cells lacking the hTLR4/MD-2 receptor complex, were used as a control in all hTLR4/MD-2 activation assays. The strains KPM318, KPM335 and KPM404 as analyzed by challenging HEK-Blue hTLR4 cells with an increasing number of colony forming units (cfu) of up to 10^6^ cfu/ml were virtually free of hTLR4/MD-2-stimulating activity, whereas their parental strains BW30270 and BL21 (DE3) elicited a substantial hTLR4/MD-2 activation already at 10^3^ cfu/ml (Figures 5A, B, 6A and B). When the extracted LPS of the strains was subjected to the TLR4-specific assay, we could confirm the lack of endotoxic activity of the samples isolated from KPM318, KPM335 and KPM404 (Figures 5C, D, 6C and D). The data also demonstrated that palmitoylation of lipid IV_A_ (when PagP was expressed) and/or modification of lipid IV_A_ with one or two P-EtN groups (when EptA was expressed) in KPM396, KPM400 and KPM402 are capable of conferring certain hTLR4/MD-2 stimulatory activity on the otherwise endotoxically inactive tetraacylated lipid A precursor (Figure 6). Our results allow us to draw the major conclusion that inactivation of regulated lipid IV_A_ modifications as demonstrated herein to be present in BL21 (DE3)-based intermediate mutant strains is a crucial prerequisite to yield consistently endotoxin-free *E. coli* strains.

**Figure 7:**
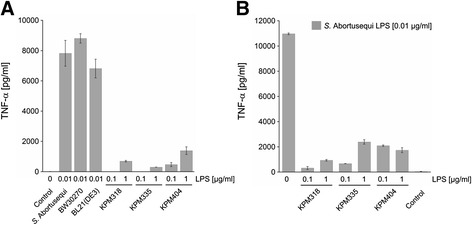
Biological activity of LPS from KPM mutants in human macrophages. Macrophages were differentiated from human blood monocytes of healthy donors. On day seven of differentiation, macrophages were seeded at 1 × 10^5^ cells/well and stimulated with LPS at the indicated amounts (LPS from *S.* Abortusequi, BW30270 and BL21 (DE3) at 0.01 μg/ml; LPS from strains KPM318, KPM335 and KPM404 at 0.1 μg/ml and 1 μg/ml, respectively) for 4 h at 37°C **(A)**. To determine the antagonistic activity of LPS from KPM strains, macrophages were incubated with LPS samples from KPM318, KPM335 or KPM404 at 0.1 μg/ml or 1 μg/ml for 30 min at 37°C, followed by stimulation of the cells with 0.01 μg/ml of LPS from *S.* Abortusequi for 4 h **(B)**. Cell-free supernatants were analyzed for TNF-α content by ELISA. The values represent the means and standard deviations from three independent experiments using cells from different donors.

As one of the critical mediators induced in response to endotoxin, we assayed the release of TNF-α upon stimulation of human macrophages by lipid IV_A_ samples of KPM318, KPM335 and KPM404. The samples exhibited very low biological activity as demonstrated by their low capacity to provoke TNF-α production in human macrophages even at concentrations of 0.1-1 μg/ml (Figure 7A). Compared to the LPS from the parental strains, which induced maximal TNF-α release at 0.01 μg/ml, TNF-α induction was reduced by about 80-95% even at 100-fold higher levels of mutant extracts. The well-documented ability of lipid IV_A_ to act as an antagonist for the hTLR4/MD-2 signaling pathway [35,36] prompted us to examine the inhibition of the agonistic activity of S-form LPS from *Salmonella enterica* subspecies *enterica* serovar Abortusequi (*S.* Abortusequi) by lipid IV_A_ samples from KPM318, KPM335 and KPM404. As shown in Figure 7B, pre-exposure of macrophages to 0.1 μg/ml and 1 μg/ml of the lipid IV_A_ extracts resulted in 72.0 ± 11.2% and 75.9 ± 2.0% (mean percent inhibition ± SD) inhibition of TNF-α production induced by LPS from *S.* Abortusequi, respectively. Thus, lipid IV_A_ from KPM318, KPM335 and KPM404 displayed potent antagonistic activity against biologically active wild-type LPS.

### Endotoxin-free expression of ApoA-1 and Hsp70

Previous studies have shown that a BL21 (DE3) ∆*lpxM::cat* mutant synthesizing a non-myristoylated LPS can be used to express heterologous proteins with reduced stimulatory activity in human LPS-responsive cells [37]. To test the ability of KPM318, KPM335 and KPM404 to serve as hosts for the production of endotoxin-free recombinant proteins, we selected the heterologously expressed human proteins ApoA-1 and Hsp70 as model systems. ApoA-1, a major component of high-density lipoprotein and important mediator in maintaining cholesterol homeostasis [38], is particularly challenging because the 28-kDa protein is known to be directly involved in neutralization of LPS toxicity and, therefore, difficult to separate from endotoxic activity [39,40]. Yet another challenging protein able to associate with LPS is Hsp70 [41]. Moreover, the 70-kDa molecular chaperone has been suggested to function as an endogenous damage-associated molecular pattern protein for activation of the TLR4 signaling pathway upon tissue injury [42]. As Hsp70-induced stimulation of innate immune system cells in many respects resembles the effects of LPS, removal of endotoxin contamination, as frequently present in recombinant Hsp preparations [43], remains a key issue to discriminate between LPS- and Hsp-induced effects.

**Figure 8:**
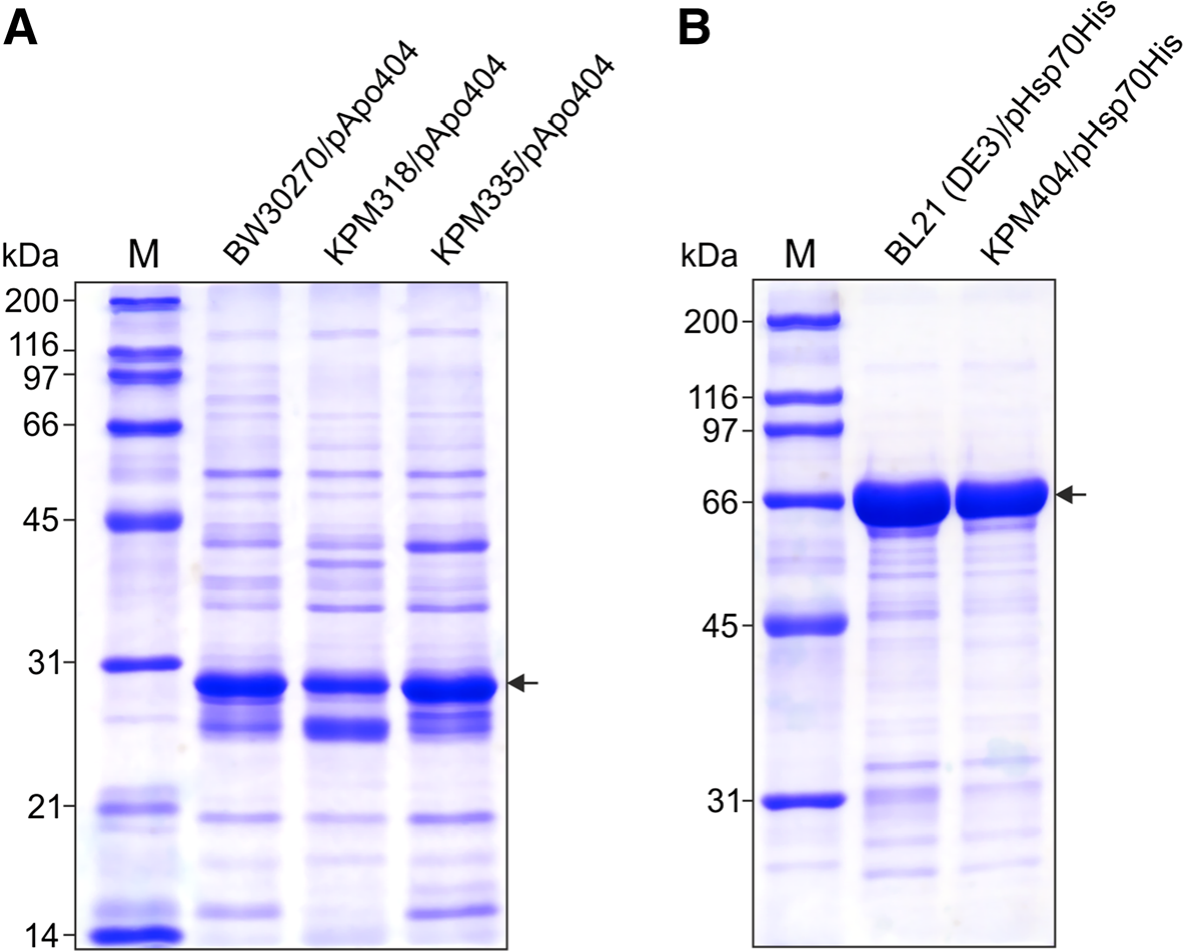
SDS-PAGE gels of ApoA-1 and Hsp70. The proteins were expressed in endotoxin-free derivatives of *E. coli* strains BW30270 **(A)** and BL21 (DE3) **(B)**, respectively, and minimally purified using IMAC on HisTrap HP (1 ml) columns. The recombinant ApoA-1 and Hsp70 samples (6 μg each) were resolved under denaturing conditions using 12% and 10% polyacrylamide gels, respectively. Molecular mass protein markers (kDa) were run in lanes M. Arrows indicate the positions of ApoA-1 and Hsp70.

For production of ApoA-1, we used the T5 promoter-based plasmid pApo404 in *E. coli* strains BW30270, KPM318 and KPM335, whereas Hsp70 was expressed from pHsp70His under the control of a T7 promoter in BL21 (DE3) and KPM404. SDS-PAGE analysis of the protein samples following minimal purification of each soluble protein extract, by immobilized metal affinity chromatography (IMAC), revealed that the endotoxin-free strains KPM318/pApo404, KPM335/pApo404 and KPM404/pHsp70His produced recombinant ApoA-1 and Hsp70 in approximately equal quantities and had similar impurity profiles as their parental strains, respectively (Figure 8). Since ApoA-1 has been described to also associate with proteins of the host cell [44], it was not surprising to detect relatively high levels of protein contaminants in the IMAC-purified ApoA-1 samples. Regardless, we did not perform any further protein purification or endotoxin removal steps to investigate the biological activity of the ApoA-1 and Hsp70 samples.

**Figure 9:**
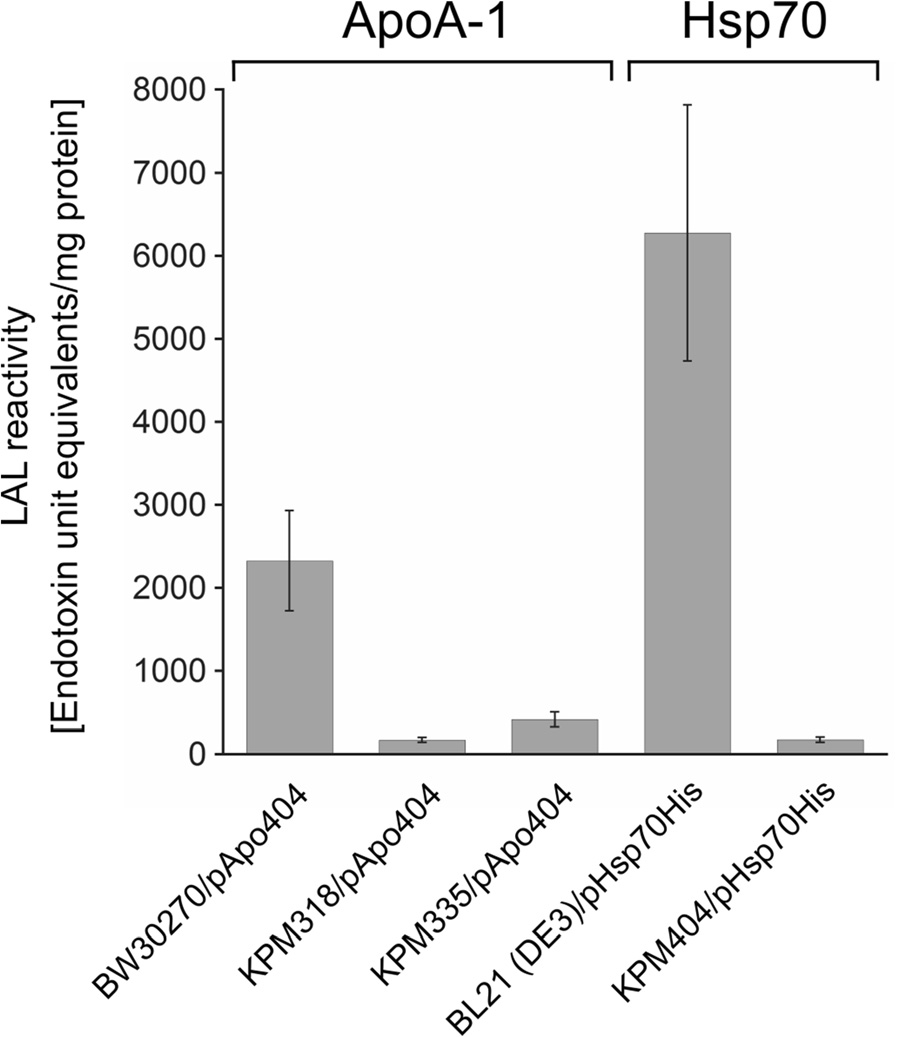
Reactivity of ApoA-1 and Hsp70 in the *Limulus* amebocyte lysate (LAL) assay. ApoA-1 was produced in *E. coli* strain BW30270/pApo404 and its endotoxin-free derivatives KPM318/pApo404 and KPM335/pApo404, whereas Hsp70 was obtained from KPM404/pHsp70His and its parental strain BL21 (DE3)/pHsp70His. The proteins were minimally purified by IMAC and assayed with the LAL test. The measurements represent the means and standard deviations from three individual experiments.

The first test of biological activity employed was the *Limulus* amebocyte lysate (LAL) assay, an FDA-approved method that is based on activation of a coagulation cascade in the LAL by trace amounts of endotoxin [45]. In this assay, the endotoxin unit equivalents determined in ApoA-1 samples from KPM318/pApo404 and KPM335/pApo404 were significantly reduced by 92.7 ± 1.3% and 82.2 ± 3.9% (mean percent inhibition ± SD) compared to those found in the ApoA-1 sample from BW30270/pApo404, respectively (Figure 9). Furthermore, when KPM404/pHsp70His was used as a host for production of Hsp70, the LAL response to the protein decreased by 97.2 ± 0.5% in comparison to the response elicited by Hsp70 obtained from BL21 (DE3)/pHsp70His. The LAL assay, though widely used for the detection and quantitation of endotoxins, is an inappropriate method to discriminate between endotoxically active hexaacylated LPS and endotoxically inactive tetraacylated lipid IV_A_ due to the presence of the LAL-activating 4′-monophosphoryl-diglucosamine backbone in both lipid structures [46,47]. As such, the LAL clotting cascade is activated by a wider spectrum of LPS/lipid A variants than LPS-responsive cells of the human immune system. The residual LAL reactivity of the proteins from the lipid IV_A_ host strains most likely reflects the non-specific nature of the assay, giving rise to false positive endotoxic results.

**Figure 10:**
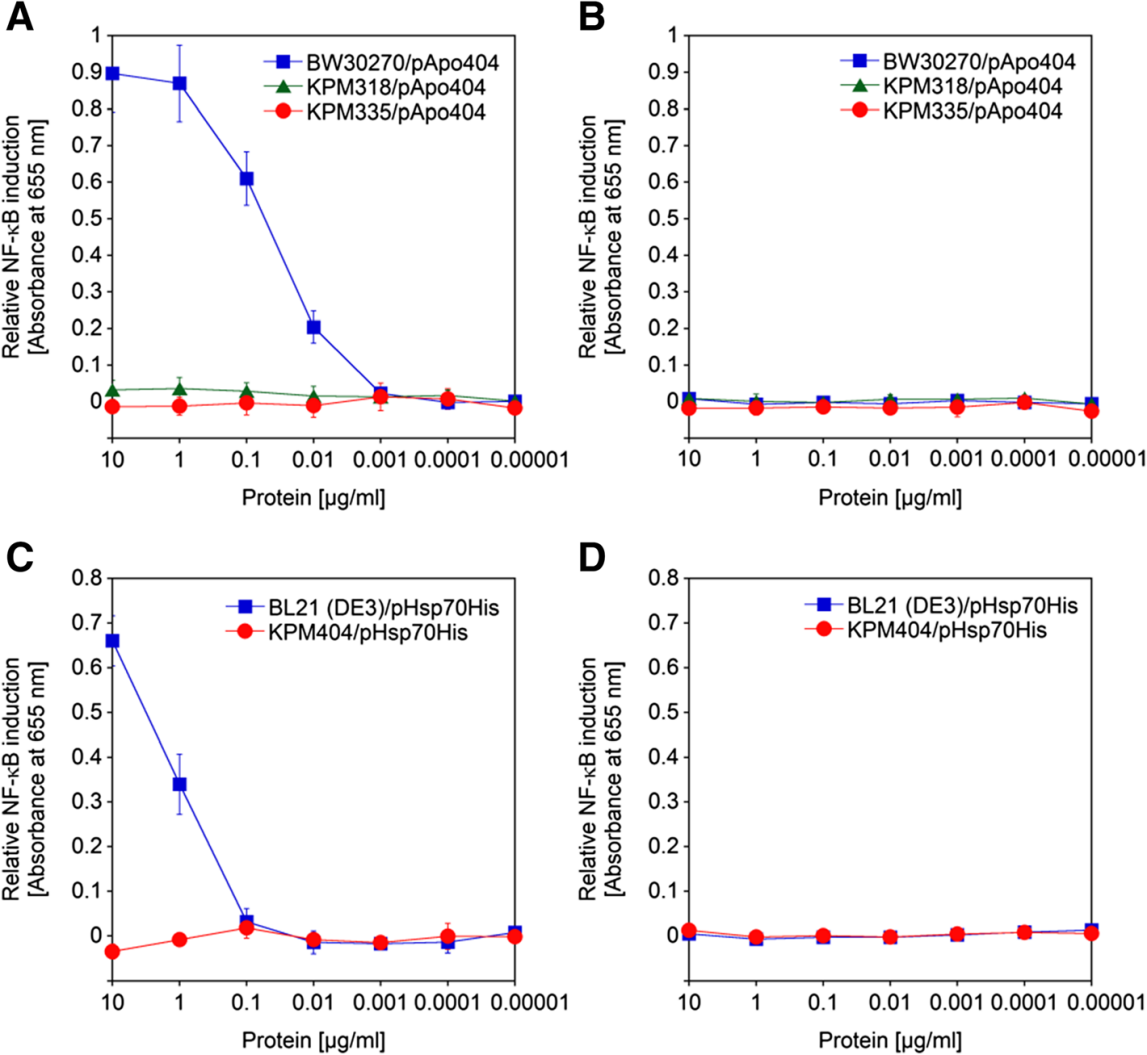
Stimulation of hTLR4/MD-2 by ApoA-1 and Hsp70 produced in endotoxin-free *E. coli* strains. The proteins were minimally purified by IMAC and assayed with HEK-Blue hTLR4 cells for their ability to activate NF-κB-dependent SEAP expression **(A** **and** **C)**. HEK-Blue Null2 cells served as a control **(B** **and** **D)**. Relative NF-κB induction was measured following stimulation of HEK-Blue hTLR4 and Null2 cells with tenfold serial dilutions of ApoA-1 **(A** **and** **B)** and Hsp70 **(C** **and** **D)** samples obtained by heterologous expression in BW30270/pApo404, KPM318/pApo404 and KPM335/pApo404, and BL21 (DE3)/pHsp70His and KPM404/pHsp70His, respectively. The values represent the means and standard deviations from three individual experiments. In all experiments, the ApoA-1 and Hsp70 samples did not activate NF-κB-dependent SEAP expression in HEK-Blue Null2 cells, suggesting that NF-κB-dependent SEAP expression was due to specific activation of the hTLR4/MD-2 signaling pathway in HEK-Blue hTLR4 cells.

In order to specifically address the endotoxic activity of the ApoA-1 and Hsp70 samples, we utilized the HEK-Blue hTLR4/MD-2 cell activation assay. The ApoA-1 and Hsp70 samples derived from the endotoxin-free strains did not trigger an endotoxic response in HEK-Blue hTLR4 cells, even when present in the assay at 10 μg/ml, whereas the proteins produced in the parental strains showed substantial NF-κB activation already at concentrations in the range between 0.1 μg/ml and 1 μg/ml (Figure 10). These results were in excellent agreement with the inability of KPM318, KPM335 and KPM404 cells and LPS to stimulate the hTLR4/MD-2 signaling pathway (Figures 5 and 6).

As exemplified herein by heterologous expression of ApoA-1 and Hsp70, proteins prepared from engineered *E. coli* strains are innately free of endotoxic activity in human LPS-responsive cells, consistent with earlier observations that KPM335 is also a suitable host for production of functional endotoxin-free inclusion bodies of the aggregation-prone fluorescent fusion protein VP1GFP [48]. However, it must be taken into account that lipid IV_A_ may act agonistically in other mammalian hosts such as mouse [49], Chinese hamster [50] or equine [36] cells, which reflects species-specific lipid IV_A_ sensing by the TLR4/MD-2 complex [51,52]. The application of proteins in cells other than human cells may therefore also require lipid IV_A_ depletion. Due to the lack of specificity, the LAL assay cannot be used to assess endotoxic activity but is ideally suited to detect residual lipid IV_A_ in recombinant proteins prepared from LPS-free *E. coli* strains. We are aware that we currently cannot respond to the question of whether further purification of ApoA1 and Hsp70 would result in complete loss of lipid IV_A_. However, our data on significantly decreased LAL reactivities of minimally purified ApoA1 and Hsp70 proteins from endotoxin-free bacteria give reason to assume that lipid IV_A_, being homogeneous, is much easier to remove from downstream products than mature LPS. Despite a common backbone structure, LPS is synthesized as a heterogeneous mixture of structurally related molecules that are decorated with various substituents, usually present in non-stoichiometric amounts [53]. These substitutions, which may vary significantly depending on growth conditions, contribute to considerable physico-chemical heterogeneity of LPS molecules, posing a major challenge to the development of a generally applicable method for endotoxin removal from proteins produced in common *E. coli* expression strains [5].

## Conclusions

Recombinant proteins manufactured in *E. coli* are inherently contaminated with endotoxin. In light of the enormous diversity of recombinant proteins expressed in *E. coli*, none of the purification methods are universally applicable to significantly reduce the endotoxin content. Instead of removing endotoxin from the protein samples, the work presented here demonstrates that it is now possible to eliminate endotoxin at the source by producing proteins in an endotoxin-free environment using LPS-free derivatives of *E. coli* K-12 and BL21 (DE3) strains. These non-conditional mutants clearly lack hTLR4/MD-2 signaling pathway agonists yet can retain viability through predominantly synthesizing the tetraacylated, endotoxically inactive lipid A precursor lipid IV_A_. The design of the strains also prohibits them from easily regaining the potential to synthesize normal LPS or endotoxically active lipid IV_A_ derivatives through acquiring mutations. This has been accomplished by incorporating a total of seven non-reverting genetic deletions that disrupt Kdo biosynthesis and prevent lipid IV_A_ from being modified with enzymes of both the constitutive and the regulated LPS pathway, while the compensating mutations *msbA52* and *msbA148* enable the *E. coli* K-12- and BL21 (DE3)-derived cells to maintain viability, respectively. The derivation of *E. coli* strains with dramatically modified LPS affords the unique opportunity to produce endotoxin-free recombinant proteins suitable for downstream experiments with human cells. These strains allow researchers to save time-consuming cleanup steps that may affect yield and functionality of the end product.

## Methods

### Bacterial strains, plasmids and growth conditions

All strains and plasmids used in the present study are described in Additional file 4: Table S4. The bacteria were grown aerobically with shaking (220 rpm) at 37°C in standard LB-Miller medium containing 10 g/l of NaCl to maintain a non-mucoid phenotype in ∆Kdo strains [54]. To induce LPS biosynthesis and restore susceptibility of Kdo-depleted KPM strains to phage P1*vir*, the media was supplemented with 15 μM d-arabinose 5-phosphate and 10 μM d-glucose 6-phosphate [11,12]. Ampicillin (100 μg/ml), carbenicillin (200 μg/ml), kanamycin (30 μg/ml) or chloramphenicol (30 μg/ml) was added to the media as required.

### DNA manipulations

Chromosomal deletions were constructed with the phage λ Red recombinase system [55] using the plasmids pKD4 and pKD3 as templates for amplification of the kanamycin and chloramphenicol resistance cassettes, respectively (for targeting cassette primers see Additional file 2: Table S2). To prevent insertion of the targeting cassettes into scar sequences of previously deleted genes, the insert cassettes targeting the *lpxM*, *pagP*, *lpxP* and *eptA* genes were generated first in separate donor strains and then transferred successively to the KPM strains by P1*vir* transduction according to standard protocols [56]. The P1*vir* donor strains were constructed by the phage λ Red recombinase procedure, except strain MWB06 was generated using the “Gene doctoring” method [57]. The kanamycin resistance cassette targeting the *lpxM* gene of BL21 (DE3) was amplified from pDOC-K with targeting cassette primers DOCBL21lpxMH1 and DOCBL21lpxMH2, digested with *Eco*RI/*Spe*I and ligated into the *Eco*RI/*Spe*I sites of pDOC-C to yield pDOC*lpxM*::*kan*. The *lpxM* gene was then replaced with *lpxM*::*kan* by co-transformation of BL21 (DE3) with pDOC*lpxM*::*kan* and the recombineering plasmid pACBSCE as described [57]. Unmarked deletion mutants were obtained by excision of the antibiotic resistance markers in the presence of the FLP recombinase encoded either by pCP20 [55] or pFLP2 [58], followed by curing of the helper plasmids at 37°C or sucrose selection, respectively. All strains were tested for loss of the relevant genes by PCR (for control primers see Additional file 2: Table S2). The nonconditional suppressor strain KPM22 L11 maintaining the normally lethal ∆*gutQ* and ∆*kdsD* mutations [12] was used as the parent for construction of KPM strains with the *E. coli* K-12 genetic background.

Strain KPM318, showing temperature-sensitive growth defects at temperatures above 40°C, was adapted to elevated temperatures by continued propagation of the bacterial cells at 42°C, which yielded strain KPM335 capable of sustained growth at 42°C.

To convert *E. coli* BL21 (DE3) into a nonconditional suppressor strain able to tolerate null mutations in essential Kdo pathway genes, the wild-type *msbA* gene of the strain was replaced by the *msbA148* allele, which was previously identified as a suppressor of the lethal phenotype associated with Kdo depletion in *E. coli* KPM22 L1 [12]. Using the primers 5ECycaI and 3ECycaQ (Additional file 2: Table S2), *msbA148* was amplified from the *E. coli* KPM22 L1 suppressor strain. A kanamycin resistance cassette targeting the essential *msbA* wild-type gene was generated as reported [12] and inserted into the chromosome of BL21 (DE3) containing the temperature-sensitive helper plasmids pKD46 [55] and pMAK705-ECmsbA. The latter plasmid was constructed by amplification of the *msbA* gene of *E. coli* BW30270 with primer pair 5HindIIIECmsbA/3BamHIECmsbA (Additional file 2: Table S2), digestion of the PCR product with *Hind*III/*Bam*HI and cloning into the *Hind*III/*Bam*HI sites of pMAK705 [59]. After transformation of *E. coli* BL21 (DE3) ∆*msbA::kan*/pKD46/pMAK705-ECmsbA with *msbA148*, screening for replacement of the ∆*msbA::kan* cassette by the suppressor allele was performed at 37°C to select for transformants that retained viability upon loss of the functional copy of the *msbA* wild-type gene encoded on the temperature-sensitive plasmid pMAK705-ECmsbA. The successful insertion of *msbA148* was then verified by PCR amplification and sequence analysis. As a result, the strain *E. coli* MWB03 was obtained, which subsequently served as the host for deletion of Kdo/lipid A genes.

The plasmid pApo404 carrying the synthetic gene with optimized *E. coli* codon usage for expression of human ApoA-1 as a C-terminally histidine-tagged fusion protein was manufactured by DNA2.0, Menlo Park, CA. The codon-optimized gene for human Hsp70 was synthesized by GenScript, Piscataway, NJ, digested with *Nde*I and *Hind*III, and ligated into the *Nde*I/*Hind*III sites of pET-22b to yield plasmid pHsp70His.

### Whole-genome sequencing and data analysis

The genomes of strains BW30270, KPM318 und KPM335 were sequenced at the Scripps Research Institute on the Illumina HiSeq instrument after library preparation with a customized protocol. Genomic DNA samples were sheared to a size range between 200 and 300 bp using an S2 Covaris ultrasonicator. The fragments were then end repaired, A-tailed with *Taq* polymerase, phosphorylated and ligated to standard Illumina TruSeq barcoded adapters following Illumina recommended protocols. The libraries were then PCR amplified for 12 cycles, followed by gel purification of the amplified libraries to select insert sizes between 200 and 250 bp.

For strains BL21 (DE3) and KPM404, the genomic DNAs were sequenced at the Research Center Borstel using the Illumina MiSeq sequencer, Nextera XT library preparation kits, and 500 cycle v2 sequencing kits as instructed by the manufacturer. Resulting reads were mapped to the genome of either *E.coli* K-12 MG1655 [GenBank:NC_000913.2] for strains BW30270, KPM318, and KPM335, or *E. coli* BL21 (DE3) [GenBank:CP001509.3] for strains BL21 (DE3) and KPM404, using the exact alignment program SARUMAN [60]. Single nucleotide polymorphisms and Indels were extracted from mapped reads by customized Perl scripts using a minimum coverage of ten reads and a minimum allele frequency of 75% as thresholds for detection.

The whole-genome sequence data of this study have been submitted to the NCBI Sequence Read Archive [SRA:PRJNA212553].

### LPS and lipid IV_A_ isolation and characterization

The dried biomasses of stationary phase 2-liter cultures were used to isolate either LPS by the original phenol/chloroform/light petroleum (PCP) procedure [61] or lipid IV_A_ by a modified PCP protocol [12]. For ESI FT-ICR, the samples were prepared as described previously [62]. The mass spectra were recorded in the negative ion mode using a 7-Tesla hybrid Apex Qe Instrument (Bruker Daltonics).

### Overexpression and purification of human ApoA-1 and Hsp70

Each strain was grown to an OD_600_ of 0.6 - 0.7 before the heterologous protein was expressed in the presence of 0.4 mM isopropyl-β-d-thiogalactoside for 3 h. All subsequent steps were performed at 4°C. The cells were harvested by centrifugation at 9,000 × *g* for 20 min, washed with phosphate-buffered saline, re-suspended in 45 ml of 25 mM Tris–HCl, pH 7.5, 0.1 M NaCl, and incubated with lysozyme (0.2 mg/ml) for 30 min and continuous stirring. Prior to disintegration of the cells by three passages through a French pressure cell at 20,000 psi, the suspension was supplemented with DNase I (50 μg/ml), RNase A (50 μg/ml) and Complete Protease Inhibitor Cocktail (EDTA-free) according to the recommendations of the manufacturer (Roche), followed by centrifugation of the lysate at 10,000 × *g* for 30 min to remove cellular debris. Imidazole was added to a final concentration of 10 mM before the cleared lysate was loaded onto a HisTrap HP (1 ml) column (GE Healthcare) pre-equilibrated with 25 mM Tris–HCl, pH 7.5, 0.1 M NaCl, 10 mM imidazole. The column was extensively washed with a stepwise gradient of 20 mM, 80 mM and 100 mM imidazole in 25 mM Tris–HCl, pH 7.5, 0.1 M NaCl, and the heterologously expressed protein was eluted using 500 mM imidazole in the same buffer. Fractions containing the protein were pooled and dialyzed against 25 mM Tris–HCl, pH 7.5, 0.1 M NaCl.

Protein samples were separated by sodium dodecylsulfate polyacrylamide gel electrophoresis (SDS-PAGE) [63] and visualized by staining with Coomassie Brilliant Blue R-250.

### Toll-like receptor activation assays

The stimulation assays using whole bacterial cells, extracted LPS samples and heterologously expressed human proteins were performed with HEK-Blue™ hTLR4 and HEK-Blue™ Null2 cells in accordance with the specifications of the supplier of the cell lines (InvivoGen). The HEK-Blue hTLR4 cells were grown at 37°C in a humidified atmosphere with 5% CO_2_ in Dulbecco’s modified Eagle’s medium (DMEM) containing 4.5 g/l glucose (PAA Laboratories), 2 mM l-glutamine (PAA Laboratories), 10% fetal bovine serum, PAA Clone (PAA Laboratories), 100 μg/ml Normocin™ (InvivoGen), 1 × HEK-Blue™ Selection solution (InvivoGen), and 1 × penicillin-streptomycin (Pen-Strep) solution (PAA Laboratories). The parental cell line of HEK-Blue hTLR4, HEK-Blue Null2, was grown under the same conditions in DMEM supplemented with 4.5 g/l glucose, 2 mM l-glutamine, 10% fetal bovine serum, PAA Clone, 100 μg/ml Normocin, 100 μg/ml Zeocin™ (InvivoGen), and 1 × Pen-Strep solution. When a 60-80% confluency was reached, the cells were detached in the presence of Dulbecco’s phosphate-buffered saline (DPBS) (PAA Laboratories), washed with DPBS and resuspended at a cell density of 1.4 × 10^5^ cells/ml in HEK-Blue test medium consisting of DMEM with 4.5 g/l glucose, 2 mM l-glutamine, 10% fetal bovine serum, PAA Clone, 100 μg/ml Normocin, and 1 × Pen-Strep solution. For stimulation of HEK-Blue cells, each sample (20 μl) was mixed with 180 μl of the cell suspension (25,000 cells) in one well of a 96-well plate (COS96ft - Corning 96 Flat Bottom Transparent Polystyrol) and incubated at 37°C for 20 h in a humidified atmosphere with 5% CO_2_. The supernatant of each HEK-Blue cell suspension (20 μl) was then added to 180 μl of reconstituted QUANTI-Blue™ substrate (InvivoGen) per well, followed by incubation of the samples at 37°C for 3 h. NF-κB-dependent SEAP activity was determined by reading the absorbance at 655 nm using a Tecan Infinite M200 NanoQuant Microplate Reader. The positive and negative control reactions were prepared with 25,000 HEK-Blue cells per well and assayed under the same conditions as described above. For the positive control experiments, tenfold serial dilutions of LPS from *E. coli* K-12 (InvivoGen) and recombinant human TNF-α (200 ng/well) (InvivoGen) were used. To determine the basal levels of SEAP activity, pyrogen-free water, 10 × concentrated Pen-Strep in DPBS, and 25 mM Tris–HCl, pH 7.5, 0.1 M NaCl, served as negative controls to assay LPS samples, whole bacterial cells and heterologously expressed proteins, respectively. The basal level of SEAP activity in untreated HEK-Blue cells was subtracted from the relative SEAP activity measured for each treated sample.

Whole bacterial cells were prepared for the assays from aliquots of overnight cultures. The cells were sedimented by centrifugation, washed with DPBS, re-suspended in a 10 × concentrated Pen-Strep solution in DPBS, and incubated first at 22°C for 3 h and then at 4°C overnight. On the basis of CFU counts obtained from platings of the initial overnight cultures, the Pen-Strep-treated cells were serially diluted appropriately in 10 × concentrated Pen-Strep in DPBS. Serial dilutions of LPS and protein samples were performed in pyrogen-free water and 25 mM Tris–HCl, pH 7.5, 0.1 M NaCl, respectively.

### Activation of human macrophages

Biological activity of LPS was tested on human macrophages derived from blood of healthy donors. The Ethics Committee of the University of Lübeck, Germany, approved the procedures. Monocytes were isolated, differentiated to macrophages and stimulated with LPS samples as described [64]. Briefly, MNCs were cultivated in teflon bags in RPMI medium supplemented with 200 mM l-glutamine, 100 U/ml penicillin, 100 μg/ml streptomycin, 4% heat-inactivated human serum type AB and 2 ng/ml macrophage colony-stimulating factor (R&D Systems, Wiesbaden, Germany) at 37°C for 7 days in a humidified atmosphere with 5% CO_2_. Macrophages were harvested on day 7, washed twice in serum-free RPMI, and seeded in serum-free RPMI containing 200 mM l-glutamine, 100 U/ml penicillin and 100 μg/ml streptomycin for experiments. After 4 h of stimulation with LPS, TNF-α was determined in cell-free supernatants using the human TNF-α ELISA set according to the recommendations of the manufacturer (BD Biosciences). Lyophilized LPS samples were re-suspended in pyrogen-free water at 1 mg/ml stock by sonication for 30 min and temperature-cycled twice between 4°C and 56°C. The samples were stored at least for 12 h at 4°C before biological experiments.

### *Limulus* amebocyte lysate assay

The *Limulus* amebocyte lysate (LAL) assay was performed using the Endosafe® Portable Test System (PTS) with single-use Endosafe®-PTS cartridges as specified by the supplier (Charles River Laboratories).

## Additional files

Additional file 1: Table S1. Summary of genome sequence data of *E. coli* strains BW30270, KPM318 and KPM335. Additional file 2: Table S2. Primers used in this study. Additional file 3: Table S3. Summary of genome sequence data of *E. coli* strains BL21 (DE3) and KPM404. Additional file 4: Table S4. Bacterial strains and plasmids used in this study.

## Abbreviations

ApoA-1: Apolipoprotein A-1
ESI FT-ICR: Electrospray-ionization Fourier-transformed ion cyclotron mass spectrometry
Hsp70: Heat shock protein 70
Kdo: 3-deoxy-d-*manno*-oct-2-ulosonic acid
LAL: *Limulus* amebocyte lysate
MD-2: Myeloid differentiation factor 2
P-EtN: Phosphoethanolamine
SEAP: Secreted embryonic alkaline phosphatase
TLR4: Toll-like receptor 4
u: Unified atomic mass unit
