# Supplementary material for: Detoxifying Escherichia coli for endotoxin-free production of recombinant proteins
Source: Microb Cell Fact. 2015 Apr 16;14:57. doi: 10.1186/s12934-015-0241-5 (PMC4404585; doi:10.1186/s12934-015-0241-5)
Supplement: Additional file 1: Table S1. — Summary of genome sequence data of E. coli strains BW30270, KPM318 and KPM335. [file 12934_2015_241_MOESM1_ESM.docx]

**Table S1. Summary of genome sequence data of *E. coli* strains BW30270, KPM318 and KPM335*^a^*.**

| **MG1655 reference base position** | **MG1655 reference base** | **Strain** | | | | | | | | | **Gene** | **Function** |
| --- | --- | --- | --- | --- | --- | --- | --- | --- | --- | --- | --- | --- |
|  |  | **BW30270** | | | **KPM318** | | | **KPM335** | | |  |  |
|  |  | **Type** | **Base** | **Predicted effect** | **Type** | **Base** | **Predicted effect** | **Type** | **Base** | **Predicted effect** |  |  |
| 193052 | G | None | G | None | None | G | None | SNP | T | D61Y | *frr* | Ribosome recycling factor |
| 547694 | A | SNP*^b^* | G | None (E38E) | SNP | G | None (E38E) | SNP | G | None (E38E) | *ylbE* | Predicted protein of unknown function |
| 547832i1 | G | INS*^b^* | G | Frameshift | INS | G | Frameshift | INS | G | Frameshift |  |  |
| 965895 | C | None | C | None | SNP | T | P18S | SNP | T | P18S | *msbA52* | ABC family transporter, suppressor of ∆Kdo phenotype |
| 3188791 | C | None | C | None | SNP | T | S46S | SNP | T | None  (S46S) | *yqiI* | Involved in detoxification of methylglyoxal |
| 3558478 | G | DEL*^b^* | Gap | Frameshift | DEL | Gap | Frameshift | DEL | Gap | Frameshift | *glpR* | Transcriptional repressor of *glp* regulon |
| 3644838 | G | None | G | None | SNP | T | V173F | SNP | T | V173F | *gor* | Glutathione oxidoreductase |
| 3753464 | G | SNP | C | None (T357T) | SNP | C | None (T357T) | SNP | C | None (T357T) | *aldB* | Aldehyde dehydrogenase B |
| 3798895 | T | None | T | None | SNP | A | N32Y | SNP | A | N32Y | *waaY* | LPS inner core heptose II kinase |
| 3813903i1 | C | INS | C | Frameshift | INS | C | Frameshift | INS | C | Frameshift | *rph-1* | Ribonuclease PH |
| 3957957 | C | SNP | T | Unknown | SNP | T | Unknown | SNP | T | Unknown | None | No feature annotated; intergenic region between *yifN´* pseudogene and *ppiC* |
| 4333631 | A | None | A | None | SNP | G | Unknown | SNP | G | Unknown | None | No feature annotated; upstream of *eptA* |

*^a^* The table shows all variations identified by mapping of the sequence reads to the *E. coli* MG1655 genome sequence [GenBank:NC_000913.2]. Uncovered regions matching the deleted *gutQ*, *kdsD*, *lpxL*, *lpxM*, *pagP*, *lpxP* and *eptA* genes are not included. The whole-genome sequence data of this study have been submitted to the NCBI Sequence Read Archive [SRA:PRJNA212553].

*^b^* SNP, single nucleotide polymorphism; INS, insertion; DEL, deletion.
