# Supplementary material for: Detoxifying Escherichia coli for endotoxin-free production of recombinant proteins
Source: Microb Cell Fact. 2015 Apr 16;14:57. doi: 10.1186/s12934-015-0241-5 (PMC4404585; doi:10.1186/s12934-015-0241-5)
Supplement: Additional file 2: Table S2. — Primers used in this study. [file 12934_2015_241_MOESM2_ESM.docx]

**Table S2. Primers used in this study.**

| **Primer** | **Sequence** |
| --- | --- |
| **Targeting cassette primers** |  |
| ECOgutQH1 | GATCGATGTGATCATAACCGGAGAGAGCAATGAGTGAAGCGTGTAGGCTGGAGCTGCTTC*^a^* |
| ECOgutQH2 | CGGCTGGCGAAACGTCTGGGATTGAAGGATTAAATAATCCATATGAATATCCTCCTTAG |
| ECOkdsDH1 | GCGATGTTGTACTGGTTATCGCCAATACTCGTTGAATAACTGGAAACGCATTGTGTAGGCTGGAGCTGCTTCG |
| ECOkdsDH2 | GCGACGCACCTGCTTTGCTCATTGTTGTTTATCCTTGAATCTTTACACTACGGATATGAATATCCTCCTTAG |
| ECOlpxLH1 | CCCAAGTTCTCCACCGCACTGCTTCATCCGCGTTATTGGTTAACCTGGGTGTAGGCTGGAGCTGCTTCG |
| ECOlpxLH2 | CGGGCGTGTTTTAAAGCGACGGTGTAACCACATATACTGCTCTGGTGCCTATGAATATCCTCCTTAG |
| ECOlpxMH1 | GCGAATACATTCCTGAGTTTGATAAATCCTTTCGCCACCCGCGCGTGTAGGCTGGAGCTGCTTCG |
| ECOlpxMH2 | GGGATAAAGATCTTTGCGCTTATACGGCTGGATTTCGCCCGGTTTGCGTATGAATATCCTCCTTAG |
| DOCBL21lpxMH1 | ATATgaattcGCTACACTATCACCAGATTGATTTTTGCCTTATCCGAAACTGGAAAAGCGACCGGTCAATTGGCTGGAG*^b^* |
| DOCBL21lpxMH2 | ATATactagtCAAACTTGAACTTATCATCAGGCGAAGGCCTCTCCTCGCGAGAGGCTTTTAATATCCTCCTTAGTTCCTATTCC*^c^* |
| ECOpagPH1 | AGCTTTGCTATGCTAGTAGTAGATTTTTGATAAATGTTTTATGGTCACAAGTGTAGGCTGGAGCTGCTTC |
| ECOpagPH2 | TACTTATTTTAGCTATTGATTTTAAAGAAGTTACTAAAACTTCATTTGTCCATATGAATATCCTCCTTAG |
| ECOlpxPH1 | CGACATGGAATCTTCTGCGATACAACAATTCGTATCTACAGAAGGTAACTGTGTAGGCTGGAGCTGCTTC |
| ECOlpxPH2 | CATGAGGTTATTATGGCCGATTTGAGGAGGGAAAGAGTAAGAGCAGTTTGCATATGAATATCCTCCTTAG |
| ECOeptAH1 | TTAATTTTGCTTTGCGAGCATATGCGCACTTTGTTCGATGGAAACACCGTGGTGTAGGCTGGAGCTGCTTC |
| ECOeptAH2 | AATCAGTCCCTGCAATAACAGCGTATCGTCTTCAACAATCAGAATTTTCATCATATGAATATCCTCCTTAG |

**Table S2, continued.**

| **Control primers** |  |
| --- | --- |
| 5EClpxLctrl | GCGGCATGATATAGCAATTATCG |
| 3EClpxLctrl | GAAAAGATAAGCACACTAATTATGCGC |
| 5EClpxMctrl | ATTAATTAACATCCATTCGCAGCCG |
| 3EClpxMctrl | CCTACAGTTCAATGATAGTTCAACAGATTTCG |
| 5ECpagPctrl | CGGAAATAAATAGAGCAGCTATTCAG |
| 3ECpagPctrl | CACAAATGCTGTGTCGGTTAC |
| 5EClpxPctrl | AGTAGCTGAAAGCAGTCAGC |
| 3EClpxPctrl | AGTAACTTACAAGTGTCTCATATCGG |
| 5ECeptActrl | ATGTATGCGCTGAATTACAACC |
| 3ECeptActrl | AACCCTAAATCCAGTACCACC |
| 5ECgutQctrl | GTCGATAAGCTGATTACCGACGC |
| 3ECgutQctrl | GTGAAACTATTCGTCAGGCACTGG |
| 5ECkdsDctrl | GACTACAGCGTGATGTTGCTGG |
| 3ECkdsDctrl | TCGACATCGAGGATCAGCAGAC |
| **Miscellaneous primers** |  |
| 5HindIIIECmsbA | ACGCCaagcttGATGCATAACGACAAAGATCTCTCTACG*^d^* |
| 3BamHIECmsbA | TCTAGAggatccTCATTGGCCAAACTGCATTTTGTG*^e^* |
| 5ECycaI | TTACTGGCAGCAAGTGCAGG |
| 3ECycaQ | AAAAGCACCAGATATGGACTACGG |

*^a^* Homology extension regions are underlined.

*^b^* *Eco*RI site is shown in lower case letters.

*^c^* *Spe*I site is shown in lower case letters.

*^d^* *Hind*III site is shown in lower case letters.

*^e^* *Bam*HI site is shown in lower case letters.
