# Supplementary material for: Detoxifying Escherichia coli for endotoxin-free production of recombinant proteins
Source: Microb Cell Fact. 2015 Apr 16;14:57. doi: 10.1186/s12934-015-0241-5 (PMC4404585; doi:10.1186/s12934-015-0241-5)
Supplement: Additional file 3: Table S3. — Summary of genome sequence data of E. coli strains BL21 (DE3) and KPM404. [file 12934_2015_241_MOESM3_ESM.docx]

| **BL21(DE3) reference base position** | **BL21(DE3) reference base** | **Strain** | | | | | | **Gene** | **Function** |
| --- | --- | --- | --- | --- | --- | --- | --- | --- | --- |
|  |  | **BL21(DE3)** | | | **KPM404** | | |  |  |
|  |  | **Type** | **Base** | **Predicted effect** | **Type** | **Base** | **Predicted effect** |  |  |
| 146424 | A | None | A | None | SNP*^b^* | C | Q281P | *yadG* | Predicted ABC family transporter ATPase |
| 550709 | G | None | G | None | SNP | A | E54K | ECD_00513 | Conserved hypothetical protein |
| 971858 | C | None | C | None | SNP | T | P50S | *msbA148* | ABC family transporter, suppressor of ∆Kdo phenotype |
| 1121275 | A | None | A | None | SNP | T | None (T113T) | *yceJ* | Predicted cytochrome b561 |
| 1627714 | A | None | A | None | SNP | C | E123A | *rstA* | DNA-binding response regulator in two-component regulatory system with RstB |
| 4105759 | A | None | A | None | SNP | G | Unknown | None | No feature annotated; intergenic region between *nudC* and *hemE* |
| 4240808 | T | None | T | None | SNP | C | None (P74P) | *basR* | DNA-binding response regulator in two-component regulatory system with BasS |
| 4240817 | G | None | G | None | SNP | A | None (Y71Y) |  |  |
| 4240904 | G | None | G | None | SNP | T | None (A42A) |  |  |
| 4240945 | C | None | C | None | SNP | T | G29S |  |  |
| 4240984 | A | None | A | None | SNP | G | None (L16L) |  |  |
| 4242687 | A | None | A | None | SNP | G | Unknown | None | No feature annotated; upstream of *eptA* |

**Table S3. Summary of genome sequence data of *E. coli* strains BL21 (DE3) and KPM404*^a^*.**

*^a^* The table shows all variations identified by mapping of the sequence reads to the *E. coli* BL21 (DE3) genome sequence [GenBank:CP001509.3]. Uncovered regions corresponding to the deleted *gutQ*, *kdsD*, *lpxL*, *lpxM*, *pagP*, *lpxP* and *eptA* genes are not included. The whole-genome sequence data of this study have been submitted to the NCBI Sequence Read Archive [SRA:PRJNA212553].

*^b^* SNP, single nucleotide polymorphism.
