# Supplementary material for: Detoxifying Escherichia coli for endotoxin-free production of recombinant proteins
Source: Microb Cell Fact. 2015 Apr 16;14:57. doi: 10.1186/s12934-015-0241-5 (PMC4404585; doi:10.1186/s12934-015-0241-5)
Supplement: Additional file 4: Table S4. — Bacterial strains and plasmids used in this study. [file 12934_2015_241_MOESM4_ESM.docx]

**Table S4. Bacterial strains and plasmids used in this study.**

| **Strain or plasmid** | **Genotype or description** | **Source or reference** |
| --- | --- | --- |
| **Strains** |  |  |
| BW30270 | *E. coli* K-12 MG1655 F^–^ *rph*^+^ *fnr*^+^ | *E. coli* Genetic Stock Center (CGSC#7925) |
| BL21 (DE3) | *E. coli* B F^–^ *ompT hsdS*_B_(r_B_^–^ m_B_^–^) *gal dcm lon* λ(DE3 [*lacI lac*UV5-T7 gene 1 *ind1 sam7 nin5*]) | Novagen |
| KPM22 | BW30270 Δ*gutQ* Δ*kdsD* *yhjD400* | [1,2] |
| KPM22 L1 | BW30270 Δ*gutQ* Δ*kdsD* *msbA148* | [1] |
| KPM22 L11 | BW30270 Δ*gutQ* Δ*kdsD* *msbA52* | [1] |
| KPM274 | KPM22 Δ*eptA::kan*; P1*vir* donor strain to generate KPM317 and KPM403 | This study |
| KPM280 | KPM22 Δ*lpxM::kan*; P1*vir* donor strain to generate KPM300 | This study |
| KPM290 | KPM22 L11 Δ*lpxL::kan* | This study |
| KPM296 | KPM22 L11 Δ*lpxL* | This study |
| KPM300 | KPM22 L11 Δ*lpxL* Δ*lpxM::kan* | This study |
| KPM303 | KPM22 L11 Δ*lpxL* Δ*lpxM* | This study |
| KPM310 | KPM22 L11 Δ*lpxL* Δ*lpxM* Δ*pagP::kan* | This study |
| KPM312 | KPM22 L11 Δ*lpxL* Δ*lpxM* Δ*pagP* | This study |
| KPM314 | KPM22 L11 Δ*lpxL* Δ*lpxM* Δ*pagP* Δ*lpxP::kan* | This study |
| KPM316 | KPM22 L11 Δ*lpxL* Δ*lpxM* Δ*pagP* Δ*lpxP* | This study |
| KPM317 | KPM22 L11 Δ*lpxL* Δ*lpxM* Δ*pagP* Δ*lpxP* Δ*eptA::kan* | This study |
| KPM318 | KPM22 L11 Δ*lpxL* Δ*lpxM* Δ*pagP* Δ*lpxP* Δ*eptA* | This study |
| KPM335 | Temperature-resistant KPM318 derivative; *frr181* | This study |
| KPM380 | MWB03 ∆*lpxL::cat* | This study |
| KPM386 | MWB03 ∆*lpxL* | This study |
| KPM391 | MWB03 ∆*lpxL* ∆*kdsD::kan* | This study |
| KPM393 | MWB03 ∆*lpxL* ∆*kdsD* | This study |
| KPM395 | MWB03 ∆*lpxL* ∆*kdsD* ∆*gutQ::kan* | This study |
| KPM396 | MWB03 ∆*lpxL* ∆*kdsD* ∆*gutQ* | This study |

**Table S4, continued.**

| KPM397 | MWB03 ∆*lpxL* ∆*kdsD* ∆*gutQ* ∆*pagP::kan* | This study |
| --- | --- | --- |
| KPM398 | MWB03 ∆*lpxL* ∆*kdsD* ∆*gutQ* ∆*pagP* | This study |
| KPM399 | MWB03 ∆*lpxL* ∆*kdsD* ∆*gutQ* ∆*pagP* ∆*lpxP::kan* | This study |
| KPM400 | MWB03 *msbA148* ∆*lpxL* ∆*kdsD* ∆*gutQ* ∆*pagP* ∆*lpxP* | This study |
| KPM401 | MWB03 ∆*lpxL* ∆*kdsD* ∆*gutQ* ∆*pagP* ∆*lpxP* ∆*lpxM::kan* | This study |
| KPM402 | MWB03 ∆*lpxL* ∆*kdsD* ∆*gutQ* ∆*pagP* ∆*lpxP* ∆*lpxM*) | This study |
| KPM403 | MWB03 ∆*lpxL* ∆*kdsD* ∆*gutQ* ∆*pagP* ∆*lpxP* ∆*lpxM* ∆*eptA::kan* | This study |
| KPM404 | MWB03 ∆*lpxL* ∆*kdsD* ∆*gutQ* ∆*pagP* ∆*lpxP* ∆*lpxM* ∆*eptA* | This study |
| MWB01 | BW30270; Δ*pagP::kan*; P1*vir* donor strain to generate KPM310 | This study |
| MWB02 | BW30270; Δ*lpxP::kan*; P1*vir* donor strain to generate KPM314 | This study |
| MWB03 | BL21 (DE3) *msbA148* | This study |
| MWB04 | BL21 (DE3) Δ*pagP::kan*; P1*vir* donor strain to generate KPM397 | This study |
| MWB05 | BL21 (DE3) Δ*lpxP::kan*; P1*vir* donor strain to generate KPM399 | This study |
| MWB06 | BL21 (DE3) Δ*lpxM::kan*; P1*vir* donor strain to generate KPM401 | This study |
| **Plasmids** |  |  |
| pKD46 | Amp^R^; λ Red recombinase expression plasmid | [3] |
| pKD4 | Amp^R^, Kan^R^; template plasmid for kanamycin resistance targeting cassette | [3] |
| pKD3 | Amp^R^, Cm^R^; template plasmid for chloramphenicol resistance targeting cassette | [3] |
| pDOC-K | Amp^R^, Kan^R^; template plasmid for kanamycin resistance targeting cassette | [4] |
| pDOC-C | Amp^R^; targeting cassette donor plasmid for λ Red mediated recombination | [4] |

**Table S4, continued.**

| pDOC*lpxM*::*kan* | Amp^R^, Kan^R^; pDOC-C carrying the *lpxM*::*kan* targeting cassette | This study |
| --- | --- | --- |
| pACBSCE | Cm^R^; λ Red recombinase and  I-*Sce*I expression plasmid | [4] |
| pCP20 | Amp^R^, Cm^R^; FLP recombinase expression plasmid | [3] |
| pFLP2 | Amp^R^, FLP recombinase expression plasmid | [5] |
| pMAK705 | Cm^R^; temperature-sensitive cloning vector | [6] |
| pMAK705-ECmsbA | Cm^R^; pMAK705 carrying the *msbA* wild-type allele | This study |
| pApo404 | Amp^R^; T5 promoter-based vector pJexpress404 for expression of ApoA-1 as a C-terminally His-tagged fusion protein | DNA2.0 |
| pET-22b | Amp^R^; T7 expression vector for C-terminal His-tag fusions | Novagen |
| pHsp70His | Amp^R^; pET-22b carrying the synthetic gene for human Hsp70 | This study |

**References**

1. Mamat U, Meredith TC, Aggarwal P, Kühl A, Kirchhoff P, Lindner B, Hanuszkiewicz A, Sun J, Holst O, Woodard RW: **Single amino acid substitutions in either YhjD or MsbA confer viability to 3-deoxy-d-*manno*-oct-2-ulosonic acid-depleted *Escherichia coli*.** *Mol Microbiol* 2008, **67:**633-648.

2. Meredith TC, Aggarwal P, Mamat U, Lindner B, Woodard RW: **Redefining the requisite lipopolysaccharide structure in *Escherichia coli*.** *ACS Chem Biol* 2006, **1:**33-42.

3. Datsenko KA, Wanner BL: **One-step inactivation of chromosomal genes in *Escherichia coli* K-12 using PCR products.** *Proc Natl Acad Sci USA* 2000, **97:**6640-6645.

4. Lee DJ, Bingle LE, Heurlier K, Pallen MJ, Penn CW, Busby SJ, Hobman JL: **Gene doctoring: a method for recombineering in laboratory and pathogenic *Escherichia coli* strains.** *BMC Microbiol* 2009, **9:**252.

5. Hoang TT, Karkhoff-Schweizer RR, Kutchma AJ, Schweizer HP: **A broad-host-range Flp-FRT recombination system for site-specific excision of chromosomally-located DNA sequences: application for isolation of unmarked *Pseudomonas aeruginosa* mutants.** *Gene* 1998, **212:**77-86.

6. Hamilton CM, Aldea M, Washburn BK, Babitzke P, Kushner SR: **New method for generating deletions and gene replacements in *Escherichia coli*.** *J Bacteriol* 1989, **171:**4617-4622.
